# Clinician Experiences of and Responses to the Challenges of Working with Patients in the Australian Compensation Setting

**DOI:** 10.1007/s10926-024-10232-9

**Published:** 2024-09-01

**Authors:** Alison Sim, Amy G. McNeilage, Trudy Rebbeck, Claire E. Ashton-James

**Affiliations:** 1 Sydney Medical School (Northern Clinical School), Faculty of Medicine and Health, Kolling Institute, The University of Sydney, Douglas Building, Royal North Shore Hospital, St Leonards, Sydney, 2065 Australia; 2 School of Health Sciences, Faculty of Medicine and Health, The University of Sydney, Camperdown, Sydney, Australia

**Keywords:** Workers’ compensation, Motor-vehicle accident, Insurer, Distress, Clinician, Qualitative

## Abstract

**Purpose:**

Previous research indicates that the compensation process is stressful for people with a compensable injury, contributing to poorer recovery. However, little is known about the challenges faced by clinicians who work in this setting. This study aims to qualitatively explore the experiences of clinicians delivering care to patients with compensable injuries in Australia.

**Materials and Methods:**

Semi-structured interviews were conducted with 26 experienced clinicians providing care to compensable patients in Australia. The interviews were transcribed, and data were analysed using reflective thematic analysis.

**Results:**

Participants described their work as challenging due to factors such as high patient distress, poor clinical outcomes, and high administrative load. However, they responded to these challenges in varying ways. While some reported feelings of vicarious injustice, emotional exhaustion, and self-doubt, others derived a sense of meaning, purpose, and mastery from these challenges. Clinician responses to the challenge of working with people with a compensation claim were associated with access to mentoring, continuous education and training, and a supportive workplace culture.

**Conclusion:**

Clinicians reported both positive and negative responses to the challenges of working with people with a compensable injury. Coping strategies that were associated with more positive reactions included seeking further education, mentoring, peer support. Prioritising these support systems is important for clinician wellbeing and patient outcomes.

**Supplementary Information:**

The online version contains supplementary material available at 10.1007/s10926-024-10232-9.

## Introduction

Up to 266,741 people are injured on Australian roads annually [1] and 130,195 people experience a serious injury at work [2]. Many of these injured individuals file an injury compensation claim and seek care for their injuries under the claim. However, accessing care from primary care providers is a critical issue for patients recovering from compensable injuries. Difficulty finding clinicians who are willing to treat compensable injuries is a major source of distress for people with a compensation claim and causes significant delays to care [3–6]. Compensation-related distress and delays to care are known to be associated with poorer outcomes for people with a compensation claim [7, 8]. Hence, it is important to understand reasons why people with compensation claims face difficulties accessing care.

Healthcare workforce data paints a picture of an ageing workforce and worker shortages, particularly in rural regions [9–11]. Furthermore, recent research suggests that many Australian clinicians intend to leave the profession, with 22% of physiotherapists [12] and up to 58% of nurses indicating that they intend to leave the profession within five years [13]. Research suggests that caring for patients who are highly distressed can be distressing for clinicians [14–17]. Clinicians who work with patients who are highly distressed have a higher risk of burnout [14], which has been demonstrated to contribute to staff attrition, decreased professional performance and increased use of sick leave [18–20]. In order to develop strategies to protect the welfare of healthcare workers and the sustainability of the workforces, it is important to understand the contextual factors that contribute to or mitigate the challenges of working with these patients.

The challenges of treating distressed patients may contribute to shortages in the workforce. It is also possible that clinicians face challenges working within compensable systems that contribute to a reluctance to work with compensable patients. A study conducted in Australia found that higher administrative burden, communication difficulties with insurers, complex patient presentations and low remuneration contributed to a reluctance by general practitioners to treat people with a compensation claim [21]. The doctors in the study reported a feeling that working with this cohort of patients was exhausting and stressful. Consistent with this, qualitative studies conducted in Australia and Sweden reported that providing care to people with a workers compensation claim was burdensome and stressful as they felt responsibility for detecting fraudulent claims [22, 23]. Supporting this, 49% of General Practitioners in Australia believe that they should be able to refuse to treat people with a compensation claim [24]. Given this, further research is needed to develop a more nuanced understanding of clinicians’ experiences of working with compensable patients, factors that exacerbate or mitigate the challenges of this work, and strategies that support clinicians to navigate these challenges.

The current research uses qualitative methods to explore the experiences of a multidisciplinary cohort of primary care clinicians who provide care for people who have a compensation claim in Australia due to injury on roads or at work. Based on previous research [6, 21, 22, 24], we expect clinicians to describe facing certain challenges in their work with people with a compensation claim and working within the compensation system. However, unlike previous research, we aim to explore the nature of clinicians’ experiences working with people with a compensation claim, and to describe the quality of their individual responses to these experiences.

## Methods

### Study Design

To understand the quality of individual responses rather than capturing the average of clinician experience, qualitative methodology was chosen. Specifically, we conducted a reflexive thematic analysis using Braun and Clarke’s six-phase framework [25, 26]. The study involved semi-structed interviews with clinicians who have recent experience working with people who are seeking care for an injury which was covered by a motor vehicle accident insurance scheme or workers compensation insurance scheme in Australia. Clinicians from five out of seven states in Australia were included to gain a rich and in-depth understanding of their experiences working in various different schemes.

### Sampling and Recruitment

Twenty-six clinicians were recruited through social media advertising and from the author’s databases of clinicians who are known to work with people with a compensation claim. We included participants from multiple disciplines to represent those clinicians whom claimants may have contact with during recovery. These included clinicians who work in primary care settings such as physiotherapists, osteopaths and general practitioners, as well as clinicians working in tertiary care settings such as pain clinics. We aimed to include both male and female clinicians and a mix of level of experience. Participants were also invited to recommend eligible colleagues to the study (i.e., snowball recruitment). Clinicians were only invited to participate in the study if they contributed to diversity in the participant cohort ensuring a diverse range of clinicians from a diverse range of clinical disciplines were represented in the data (i.e., purposive sampling). This included exploring opportunities to connect with experienced clinicians by asking participants and researcher networks to recommend experienced colleagues. Ethical approval was granted from The University of Sydney Human Research Ethics Committee (reference number 2021/881). All participants provided verbal consent at the beginning of the audio-recorded interview. Participants were informed that they may withdraw from the study at any time prior to the publication of results and that the results would be de-identified. Recruitment continued until it was felt that the data captured a broad range of experiences, and new perspectives and information was no longer raised in the interviews [25, 26].

### Data Collection

Interviews were conducted and recorded over Zoom between January 2022 and May 2023. Participant demographic information was collected using an online survey (Qualtrics) which was completed prior to their interview. A semi-structured interview guide including open-ended questions and prompts (see Appendix A) was used to uncover the individual experiences, attitudes, and context of participants who were treating patients with compensable injuries in Australia. The interview guide prompted participants to reveal both barriers and facilitators to providing care in this context, or factors that may shape their individual experience of treating people with a compensation claim. The interview guide was pilot tested over the first three interviews to ensure questions were easily understood, interpreted as intended, and neutral in their framing to avoid biasing participant responses. After the first three transcripts were reviewed, no changes were made to the interview guide and hence data from these pilot interviews were included in the main analysis. The first author (AS) undertook all of the interviews in order to increase depth of engagement with the data set.

Interviews were transcribed verbatim and NVivo software [27] was used to manage the data and codes during the analytic process. A reflective research journal documented the research teams’ initial thoughts and responses to the interviews as they were conducted. This helped to enhance reflexivity and directly challenge the assumptions, beliefs, and values underpinning research team members’ interpretation of interview content [28].

### Data Analysis

Braun and Clarke’s six-phase framework for reflective thematic analysis was adopted for data analysis [25, 26]. This process is well suited for analysing a rich dataset due to its systematic and flexible nature. The method allows for a thorough exploration of complex and nuanced data, enabling the researchers to identify patterns and themes across diverse information. Its iterative and recursive approach ensures a comprehensive understanding of the dataset, facilitating the extraction of meaningful insights and the development of robust themes [25, 26].

#### Phase 1: Familiarisation with the Data

The transcripts were initially reviewed by two authors (AS and AM), who immersed themselves in the data by reading and re-reading the transcripts to become thoroughly familiar with the content. This phase included noting initial observations and potential patterns.

#### Phase 2: Generating Initial Codes

Using an inductive approach, AS and AM collaboratively generated preliminary codes, identifying and labelling features across the dataset. This coding process helped to capture the essence of each element of the data.

#### Phase 3: Searching for Themes

Further transcripts subsequently underwent independent coding by AS and AM and their coded outputs were systematically compared. In this phase, the codes were collated into potential themes, organising different codes into broader patterns of meaning.

#### Phase 4: Reviewing Themes

AS then coded the remaining transcripts, adhering to the established codes and generating new codes where necessary. This phase involved reviewing and refining the themes to ensure they accurately represented the data and formed coherent patterns.

#### Phase 5: Defining and Naming Themes

The evolution of the themes from these codes was then undertaken through a collaborative and iterative process (AS, AM and CAJ). Naming the themes aimed to capture the essence of what each theme represented.

#### Phase 6: Producing the Report

Finally, the analysis was written up, weaving together the analytic narrative and data extracts to tell coherent and compelling story about the data. This phase provided an argument about the significance of the identified patterns.

### Researcher Reflexivity

Engaging with data in the pursuit of meaning through qualitative methods inevitably involves the researcher’s experiences and biases shaping the interpretation of the data. Recognising the contextual factors that impact our research allows researchers to provide transparency, aiding the reader in comprehending the specific lens through which the researcher has interpreted the data [28]. The first author (AS) works as an osteopath in chronic pain settings, frequently with people who have a compensable claim. Her own experiences in this setting as a clinician experiencing many of the elements described by the interviewed clinicians undoubtedly influenced the desire to complete this research project and contextualise her own experiences. As such, AS acknowledges she may be inclined assume that participants’ experiences, attitudes, or beliefs are akin to her own. AS recognises that she has been exposed to many clinical situations where claimants have a very difficult time dealing with both recovery from injury and the requirements of the insurance claim. There are many experiences where AS has felt that claimants were exposed to unnecessarily unfair treatment or poor handling by the insurers. She also recognises that this naturally one-sided version of events where only the claimant’s story is being represented is likely to bias a clinician to feel that insurers don’t always look out for claimants as well as they could. Authors CAJ and AM are researchers who have, in their previous research, observed a relationship between clinician exposure to people with a compensation claim and clinician burnout. Author T.R is a clinician-researcher who commonly reviews disputes arising between people with compensation claims and insurers, works as a clinical specialist with injured people with a compensation claim who do not recover well, and has conducted multiple clinical trials in this area. All interviews were completed by AS who was acquainted with several of the participants, either through professional acquaintance or in some cases as former work colleagues from a pain clinic. All participants were provided with information about the study including a brief synopsis of the intention of the study and information regarding the background of the primary researcher (AS, PhD student, clinician, funded by philanthropic grant).

## Results

### Participants

An even mix of genders was represented in the participant sample. The practice settings included private practice sole practitioners and group practice. We also included clinicians from specialist pain physiotherapy clinics and interdisciplinary pain clinics. The cohort was well represented by experienced clinicians with 12 of the 26 clinicians having over 20 years of experiences. The range of clinicians included was very broad with eight clinician professions included. 19 of the clinicians were physical medicine clinicians and 16 out of the total cohort were located in Victoria. The numbers of people with a compensation claim seen per week by clinicians was varied. The participant population was mainly Caucasian and as such, there was little ethnic diversity represented in the cohort (See Table 1).

**Table 1:** Demographic characteristics and professions of participants

Clinician participant characteristics	Total (*N* = 26)
Gender	
Female	13
Male	13
Years of experience	
1–4	2
5–10	3
11–15	6
16–19	3
20 or more	12
Geographical location (Australian state or territory)	
Victoria	16
New South Wales	4
Queensland	2
Western Australia	2
South Australia	2
Profession	
Physiotherapist	16
Osteopath	3
Sports physician	1
GP	1
Pain physician	1
Occupational therapist	2
Psychologist	1
Nurse practitioner	1
Practice settings	
Group practice primary care	10
Group practice tertiary care	1
Interdisciplinary pain clinic	8
Specialised pain physiotherapy group practice	6
Sole trader primary care	1
Number of compensable patients seen per week	
1–5	12
6–10	5
11–15	2
Unsure/NA	7

### Thematic Analysis

Five major themes that were equally evident were developed: 1. Clinicians experience vicarious injustice when exposed to negative claimant experiences, 2.Emotional Exhaustion and coping, 3. Working with clinical complexity can cause self-doubt, 4. Helping this clinical population can be meaningful, and 5.Professional mastery was nurtured through experience but stifled by systemic constraints. Table 2 provides a brief synopsis of the content of each of the themes and the clinicians who contributed to them.

**Table 2:** List of major themes

Themes	Content	Participants contributing to theme
Vicarious Injustice	Patient stigmatisation Poor treatment of patients by insurers Clinician frustration	1,2,3,5,6,7
Emotional Exhaustion	Administrative burden Emotional burden Coping strategies	4,5,7,8,9,10,11,12, 13,14,16,17,4,5,18, 19
Self-doubt	Overwhelm and helplessness Lack of training Education as buffer	5,8,7,13,17,18,19
Meaningful work	Helping people feels good Helping the system is a good use of time Opportunities for learning and collegiality	7,9,13,15,19,20
Mastery	Challenging work is rewarding Experience Education Mentoring System factors that prevent best practice	6,7,8,11,13,19

### Theme 1: Clinicians Experience Vicarious Injustice When Exposed to Negative Claimant Experiences

Clinicians caring for their patients under compensable systems often bear witness to this group of people experiencing distress. Some clinicians find the vicarious exposure upsetting and need to manage their own distress in response. Vicarious exposure to claimant distress was usually related to issues with dealing with the insurer, the claims process, and the delays that are often reported by people with a compensation claim. In the process of describing how a patient under her care was experiencing stigmatisation and dehumanisation during an insurance claim, this physiotherapist is clearly upset on behalf of her patient:

“*That puts a lot of stress on patients… they have to tell the whole story over and over again… I even had one lady who had to, you know, do like a drug [test] … she had to do a urine sample with the door open to prove that she wasn’t a drug addict. You know, it was just awful”* (Clinician #1). Another physiotherapist describes how extended delays in insurance processes impacted their patient and that this was difficult for them to observe: *“I also get angry…on behalf of the client that they’re having to go through this”* (Clinician #2).

Many clinicians will have an extended relationship with their patients and walk beside them throughout their often long and complicated recovery. This longer-term relationship often leads to a strong rapport. Observing the injustice experienced by these patients can be especially upsetting for clinicians given their investment in the wellbeing of these patients. One osteopath described the experience of caring for a long-term patient who had a particularly difficult time in the insurance system. He appears to be particularly attuned to, and upset by the patient’s experience of not feeling cared for by either his employer or the insurer:

*“One of the fellows that I’m looking after at the moment, he’s been in there [injured and under insurance claim] for two and a half years… after he was run over at work… Once he [went] back to work, no one really cared. He’s never really had a case manager… You know, he’s ended up sort of floating, longer and longer and longer. But over time that breeds a little bit of resentment. And it has sort of got now to the point where this sweet gentle giant, he’s very upset at the way that he’s been treated and the perception that his employer doesn’t care, that the insurance doesn’t care and it’s just him having to deal with it”* (Clinician #3).

Being exposed to many of these upsetting scenarios can be difficult for clinicians and, many described feeling empathy for their patients when describing their hardships. One occupational therapist, a mother herself, describes the experiences of a patient who is a single mother and having issues with getting approval for treatment in her claim:

*“The experience that I’ve had is that the clients have found it to be a very frustrating and overwhelming experience… This lady, actually I am quite worried for her mental health as she’s had roadblocks all the way through her entire claim … I actually spoke to her a few weeks ago and she was in tears”* (Clinician #2).

Similarly, this clinician describes how the frustration felt by people with a compensation claim is frequently also felt by the clinicians who are helping them:

*“I find that they are frustrated. The clinicians are frustrated. Everyone in the whole clinic is frustrated just because of the [limited] access that these patients have to the treatments that they need. I find that’s the hardest part of it.”* (Clinician #5)

Observing the frustration and distress experienced by patients often prompts clinicians to step in to try and advocate for their patients. Where sometimes this may feel helpful, it frequently takes time away from clinical work and is not always fruitful. For example, “*I’ve had a couple of times where I’ve gone in to [advocate] for people and it’s just been a bit of a waste of time, or it’s actually been quite distressing for me”* (Clinician #6).

The flow on effects of these issues observed by clinicians were patient disengagement from treatment and poorer clinical outcomes, which were upsetting for both patients and their clinicians, for example:

*“They feel quite upset and vulnerable and angry, because they haven’t been treated well in the compensable system, then they’re assuming the worst of the rest of their treating team as well. They’re assuming that because the (insurer) hasn’t provided them with timely care… then you won’t either. That can be challenging to build rapport,”* (Clinician #7).

The distress that clinicians feel from being exposed to these scenarios in patients who they care for undoubtedly contributes to the sense of emotional exhaustion described in the next theme.

### Theme 2: Emotional Exhaustion Andcoping

Clinicians working with patients under compensable care face many additional tasks compared with their work with private patients. Additionally, the patient population tends to present with much greater distress and unmet needs, frequently caused by insurance-related delays to care or other communication issues. The combination of the both the additional administrative load, including the need to deal with other stakeholders such as employers, and dealing with high patient distress frequently leads participants to contrast their experiences between treating private patients and people with a compensation claim. A sense of emotional exhaustion was frequently reported by clinicians when describing their response to the complexity of treating people with a compensation claim. For example, one physiotherapist described:

*“I would just often have this really profound sense of exhaustion, like at the end of my day, or like on my days off. I just feel like I was just sapped… I think that feeling of exhaustion is definitely something I hear from a lot of other people… who are working in similar sort of settings to me”* (Clinician #8).

Similarly, this physiotherapist reports: *“I’m certainly drained to a degree, and more so as I’ve got older. You just don’t bounce back quickly”* (Clinician #9). Another experienced female physiotherapist described being *“exhausted, and probably dreading going back next day”* (Clinician #10) after treating many compensable patients in a day*.*

The consequences of these feelings of exhaustion varied for clinicians and was influenced by many factors. For some clinicians and their teams, the additional emotional and administrative burden meant that some clinicians would refuse to see compensable patients or meant that there was a sense of stigma associated with those patients in the workplace. For example:

*“We don’t like treating people who are being covered by interstate work covers, or some of the insurance companies who are really difficult to deal with.” (*Clinician #11).

“*The clinic that I’m working at here isn’t as supportive of [compensable] patients as some places I’ve worked over the years. They typically don’t bulk bill or encourage any third-party payers and so I take on board all of that risk for my clients… the amount of time required to get paid slightly less by the [insurer] just made it untenable and we just couldn’t deal with them any longer… we made the decision together that we weren’t going to waste more time dealing with [the insurer] and that we would work with her GP and look at the Medicare plan to support her.”* (Clinician #12).

*“The culture at the clinic that I worked at was to share [compensable patients] around …The patients were treated as if they were a burden, which was really sad... [The clinic would] book them in on a rotating roster to prevent the practitioners themselves from being burnt out.”* (Clinician #7).

Coping with being exposed to claimant distress and the exhaustion it brings was a variable experience. Some clinicians felt that not getting too emotionally involved in the patient’s story helped to maintain their own emotional balance. For example: *“I don’t… have a strong emotional… response. In fact, I tend to feel that’s a little unhelpful. I tend to look at… these really complex sort of problems… with a degree of objectivity,*” (Clinician #13). Others found that allowing enough time to deal with the complex nature of the presentations, as well as knowing when to refer to other clinicians helped. For example:

*“If you have that type of particular patient, we need a little bit more time to listen to them, to really listen to what’s going on in their lives and what’s causing their stresses. And then, is it something that we can handle or something that we need to put a referral into the G.P?*” (Clinician #14).

One physiotherapist reported that his religious faith brought a reassuring perspective which helped in managing his response to claimant distress (*“I think there’s a religious perspective… I hold a Christian view, a Christian perspective on the world. I think that’s relevant for me,”* Clinician #15). Reducing the number of compensable or otherwise complex patients was reported by several clinicians as a strategy to reduce exhaustion (e.g., *“If you have too… many complex patients in one day, I couldn’t manage that myself… From a very early stage of my private practice career… I needed to diversify,”* Clinician #9).

For those clinicians who worked with large numbers of compensable patients, some reported the need for dedicated self-care strategies. For example:

*“Being able to walk away, turn off your computer, and [recognise] you can only control what you can control. You need to switch off … If you’re not switching off it does impact the quality of care.”* (Clinician #16).

*“Sometimes it might take a minute of just laying back in my chair and… take a few deep breaths or something.”* (Clinician #4).

*“Exercise and relaxation and talking to colleagues, peer mentoring.”* (Clinician #17)

*“I will go for a big walk after work, or to a Pilates work out… something to try and just de-stress*.” (Clinician # 5).

Some clinicians reported that dealing with high claimant distress, such as in the case of patients presenting with suicidal ideation, was difficult to cope with after leaving work. Some described ruminating about the patients at home (“*Taking that home. Have I said the right thing, did I say something wrong, which may contribute to this [suicidality?]”* Clinician #18; *“When you go home, thinking about what happened to that patient. Are they okay? It plays on your mind a bit, I guess, after work,”* Clinician #5), Other clinicians described different responses such as *"being quite emotionally distant from my family and just, you know, wanting a bit of solitude and space myself”* (Clinician #17) or feeling *“I need a beer after that day”* (Clinician #19). Many clinicians mentioned burnout in the context of these discussions. One clinician observed of other colleagues:

*“Sometimes people have a lot of sick leave… people do just call in sick because they can’t be bothered. They can’t deal with it. They don’t want to have to go back and do it all again another day. … I know that some people just change careers”* (Clinician #5).

This theme explored the commonly reported experience of feeling exhausted or drained when working with injured people with a compensation claim. Most clinicians reported that the interactions with distressed people with a compensation claim was a reason for this response. Many of the responses described could be seen as coping strategies for dealing with the clinician’s own distress.

### Theme 3: Self-Doubt Prompts Upskilling and Support Seeking

In the process of describing their work in these complex clinical settings, many clinicians expressed a sense of self-doubt in their abilities, particularly in being able to help highly distressed patients. At times, they would describe feeling overwhelmed by the nature of the situations they were presented with and a subsequent sense of helplessness in not being able to provide solutions. Some clinicians would describe feeling out of their depth, under prepared, un-supported or a sense that they were working outside of their scope of practice. For example:

“*As a clinician, that’s really challenging to manage because we don’t really get that training right? We get trained to manage the biological factors… But when you’re then trying to do those things in the context of someone who’s really distressed… that can become really challenging for the clinician. I don’t think we get the support and the training to know how to start to unpack that and help someone to navigate that distress so that they can actually engage in the things that we think would be beneficial.”* (Clinician #8).

When attempting to navigate insurance processes such as getting approval for treatment on behalf of a patient, clinicians described that the difficulties or failures that they experienced with these processes was upsetting. They would frequently question if it was their lack of knowledge of skills that had brought about the situations or let the patient down. For example*: “You feel helpless… Is it my application? What am I doing wrong?… You start to doubt yourself”* (Clinician #19). A physiotherapist similarly described: *“Often I’m second guessing if I am doing the right things. Is it a failure on my part? Am I missing certain elements?*” (Clinician #18).

This sense of feeling underprepared was particularly evident for some clinicians who were regularly dealing with people with a compensation claim who were expressing suicidal ideation. For example:

*“Sometimes they are… threatening to end their life, which is quite challenging when you’ve had no formal training… in that area... That’s quite stressful as a clinician [and] takes up a lot of time and resources... I feel like [clinicians] maybe need more training in that area”* (Clinician #5).

The life and death nature of these situations were often described as being the most stressful clinical experiences, with clinicians frequently feeling frightened and out of their depth. For example, *“Those high levels of distress are always the most challenging. High levels of depression or suicidal ideation which really raise the stakes of the appointments and the interactions that you have…. It just feels very scary”* (Clinician #17).

For many clinician participants in this study, the sense of feeling out of their depth encouraged them to pursue further education to fill these gaps. For example, an osteopath described: *“Because they had a lot of extra psychosocial components and they weren’t strictly musculoskeletal cases, I felt like I needed to know more. I needed to be more specialised to be able to help them”* (Clinician #7). When asked about the consequences of working in complex settings, a physiotherapist stated that: *“The consequences have been life changing. It has sent me down a pathway of wanting to know more. It has directed me into more of a psychologically informed physiotherapy approach”* (Clinician #8). Another physiotherapist described further education as a buffer for dealing with the complexities of the work: *“I do think experience and further education has helped my process*” (Clinician #13). He explained that it helped him to define his scope of practice and to identify when he was not responsible for certain issues and needed to refer or seek additional support:

*“There are issues here that I can’t deal with. So then [I did further training] and found out about the psychosocial issues as well. I feel much more confident having the background knowledge to say these issues here are beyond my control*,” (Clinician #13).

As well as seeking further education as a buffer to helplessness, some clinicians sought the support of their team (e.g., *“I feel protected by the team around me here. The specialists and other allied health team are there to speak to immediately after, which is wonderful, because… it is quite a threatening situation,”* Clinician #17). Similarly, an experienced physiotherapist described the importance of:

*“surrounding yourself with other practitioners who you can spin things off. You need to that… In a practice where there are other physios and you can either have a corridor chat about this difficult patient or hopefully you can have case presentations where you can problem solve and brainstorm”* (Clinician #10).

In this theme, clinicians described feeling unsure of their abilities, uncertain about how to proceed, and in some cases that they may have been letting down their patients. Clinician responses to these feelings included upskilling and seeking support from peers. For many of the clinicians interviewed, these experiences contributed to steps in a career that led them to develop a sense of mastery in how they delivered care in these complex settings, described further in Theme 5.

### Theme 4: Helping this Clinical Population can be Meaningful

In the process of describing the complex and often emotionally draining experiences involved in working in compensable care settings, clinicians would often contrast this by describing the factors that made the work meaningful. These explanations were perhaps provided to justify their decision to continue working in this setting despite some negative experiences, for example: *“You do have some days when you walk out and think, yes, I’ve made a real difference to people’s lives and some days when it’s the complete opposite,”* Clinician #19. Some clinicians described a curiosity about the individuals they were treating and a desire to help. They would often then go on to explain how helping others made them feel fulfilled. For example:

“*I really care about people. I’m interested in their emotions and feelings… I think it is like my inner being is, in a way, dancing with theirs a bit... I still love exactly what I do. It’s still very privileged to be doing that. It’s a bit of a joy, and even though it’s taxing, it’s something that I’ll do as long as I feel like I’m doing a good job.”* (Clinician #9).

“*I think deep down I like to help and be useful. I think facilitating people to recover, to reclaim themselves would probably be the thing I enjoy the most.”* (Clinician #13).

One physiotherapist reported that taking time to engage with and educate insurance staff, such as claims managers, was meaningful to him as it meant that future people with a compensation claim may benefit. As such, he felt this was a good investment of his time to help improve the claimant experience*: “One of the things I do see as a responsibility is to educate insurers. So, I’ll frequently find myself, as I’m discussing a particular patient with the insurer, particularly if I go in with a conciliatory tone… I have a good knowledge in this area, and I can be informative… I see that as a good way of… doing my part to educate people in the system,”* Clinician #15. A doctor explained that the work was meaningful because they were able to learn clinical tips during interactions with the doctors from the clinical panels of the insurance body. He felt that in reviewing complex cases with the insurance doctors, the suggestions that were provided for medication review were sometimes helpful in a practice setting where such case conference options are not easily available:

*“They might suggest this or that instead as an alternative. Sometimes they might even contact you out of the blue and ask ‘have you thought about this?’ It’s helpful. It depends on the skills and experience of the person calling… but it can be a more collegial sort of experience which can be very helpful*” (Clinician #20).

In being asked about working in compensable care settings, clinicians had often spent time describing the flaws of the system that impacted both themselves and their patients. However, being able to provide assistance to this vulnerable population also provided opportunities for fulfilment on a personal and professional level and a reason to get up for work in the morning.

### Theme 5:Professional Mastery was Nurtured Through Experience but was Stifled by Systemic Constraints

As well as describing that the work is meaningful, clinicians frequently expressed a sense of professional accomplishment in being able to engage with the complexity of the work, both from a psychosocial as well a systems navigation point of view. They found the challenge of being able to unpack the complexity and find creative solutions to help their patients to be rewarding and satisfying. At other times, however, clinicians would find that system factors might prevent them from being able to engage with the work on this higher level, leaving them frustrated and professionally unsatisfied.

As described previously, clinicians often feel a sense of self-doubt that might prompt further education to move them to a place where they feel more comfortable working in this setting. Combined with experience, further education was often pursued to build confidence to move a clinician to a place of mastery in their field. For example:

*“My initial upskilling sort of came from this feeling of oh, my God! What do I do for these people? I need to equip myself in some way to know how I can actually help them because what I’m doing is not working… These days I feel a lot more equipped to deal with it. I enjoy the challenge of being able to help people who really need it.”* (Clinician #7).

*“I’ve gone through postgraduate then specialisation training, more for my development and self-interest… I gravitated towards [this type of work] because it’s more of a challenge.”* (Clinician #13).

Another clinician described how the complexity of the work kept her feeling professionally challenged and allowed her to use all her skills and experience to unpack difficult scenarios:

*“I found personally, my brain doesn’t do well with very simple or linear sort of tasks. I very much enjoy that sort of complexity and situations where there’s lots of factors interacting. It sort of almost requires that bit more of an abstract approach to seeing those interactions and those interplays and, you know, sort of almost coming up with…creative solutions.” (*Clinician #8*).*

Clinician (#8) went on to describe that this brought personal and professional satisfaction that offset the negatives of working in this space: *“It’s almost like a bit of an art process… It’s that really collaborative approach to what is actually going to be workable for you, and that’s the bit I think that really draws me in. I really enjoy and love it,” Clinician #8.* A physiotherapist also described the joy that he experienced from using his skills and experience to get a good outcome in his work:

*“The enjoyable bits, the discovery, and particularly the discovery for them… The detective part of that. The linking and building a sense of confidence is really huge and rewarding. Because wow! I think I’ve really done something here, you know, facilitated something here for this [person] to really get back and be really happy again… I love that. I love that. What could be nicer than that?”* (Clinician #9).

As well as gaining skills and experience to help patients presenting with complex psychosocial factors, clinicians explained that knowing how to navigate the insurance system brought with it a sense of confidence in being able to assist patients to do well within the system. One clinician described this as *“knowing how to play the gam*e” (Clinician #6). Another element of mastery described by clinicians was being comfortable with ambiguity, whether it was for patient factors such as diagnosis or prognosis, treatment planning, or navigation of insurance processes such as challenging denied treatment requests. An acceptance of the flaws of the system also seemed to help clinicians to roll with difficulties and buffer against feelings of helplessness. For example:

*“That’s changed across [my] career. I think I’ve got to a pretty comfortable place where I’m comfortable with ambiguity. I don’t expect that I need to know everything because I don’t think that’s plausible. And so, I think I feel more satisfied… You’ve done what you think you can do. You’ve put your effort into it. You’re not going to get much more if you invest much emotionally or professionally. I can let it go.”* (Clinician #13).

In supporting the development of mastery, working in a supportive team and having good mentors was reported to be important. One physiotherapist explained that her workplace placed a great deal of emphasis on mentoring, knowing that without helping junior clinicians to understand the nuances of working in complex care settings, they may leave or experience burnout symptoms*:*

*“We do a lot of training for communication and framing of tricky conversations… Trying to make sure that they understand where each of those key stakeholders is coming from and their perspectives. We do a lot of modelling, and so, we call them doubles in the clinic. Observe that behaviour that we perceive to be really good… those kinds of things help.”* (Clinician #6).

Other clinicians described how mentoring helped them to place a different emphasis on what they saw as a successful clinical outcome in complex settings, which helped to improve professional satisfaction. For example:

“*when [a treatment] went well, I [felt like] the best physiotherapist ever. If it went badly, [I felt] like the worst clinician and that impostor syndrome really started to rear its head. So, riding the outcome was something that I did early, and wasn’t great… In mentoring with more experienced clinicians, you know, they started to reframe what a good outcome looks like… it helps you to reframe what your role is*.” (Clinician #11).

At times, the drawbacks of working with insurance systems, particularly those that led to delays to care, interfered with clinicians being able to achieve a sense of mastery in their work. For example:

*“It’s demoralising because, you know, you’re getting into healthcare, hoping that you’re going to make a difference and then you’ve got clients in front of you and you’ve got all your wealth of experience, and you feel like you can offer a lot to these clients’ lives, and yet you’ve got your hands tied behind your back because it is pending feedback communication [slow communication from the insurer is delaying approvals for treatment]*.” (Clinician #19).

Similarly, other clinicians explained that the constraints of the system meant that they were unable to deliver care in a way they felt was best practice. As such, they felt they were put in a situation where they were delivering substandard care. This often created internal conflict for the clinician. For example:

*“It’s a bit of an ethical thing for the clinician because you then go well, how much do I bend the rules of the system under which this person is being funded in order to deliver what I think is person-centred care? Versus, do I compromise on that person-centred care in order to make the requirements for me practicing and delivering a service under that model? So, I think there’s some really sort of challenging ethical and moral things happening there.”* (Clinician #8).

Clinicians, particularly those with further education and many years of experience, were easily able to describe a sense of mastery and confidence in their work that made dealing with these complex clinical scenarios challenging and rewarding. On the other hand, where the systems that create the complexity in the first place became too complex and intrusive, clinicians frequently described finding themselves hamstrung and unable to use these high-level skills to benefit their patients.

## Discussion

Consistent with previous research [6, 21, 22], clinicians in this qualitative study reported that many aspects of working with patients within the compensation setting are challenging, including patient complexity and administrative burden. Clinicians responded to these challenges in different ways: While some reported experiencing and observing other clinicians experiencing self-doubt and vicarious injustice that left them feeling emotionally exhausted. Others responded to these challenges by leaning into peer support from colleagues and seeking out additional training. Those clinicians who felt well supported by their peers or mentors, or who sought further training, reported that their work with compensable patients was fulfilling and meaningful, giving them an opportunity to extend themselves clinically, and experience a sense of mastery and accomplishment. These findings suggest that working with compensable patients can be challenging, but that further education/training and peer support or mentoring may protect clinicians from stress and burnout, and foster wellbeing at work.

### Vicarious Injustice

Clinicians in this study described experiencing vicarious injustice in response to hearing their patients describe unjust treatment in compensation processes. The concept of vicarious injustice has been described previously in organisational settings; for example, where employees observe unfair treatment of their co-workers [29, 30]. Research suggests that observers of interpersonal injustice are more likely to have a strong negative reaction if a) they interpret the perpetrator’s intent to cause harm [31] and b) they empathise strongly with the victim [32]. This would suggest that clinicians who believe that the intentions of the compensation system are to be helpful rather than harmful, are less likely to experience vicarious injustice than those who doubt the intention of the insurer. Vicarious injustice is described as a distressing experience; however it may serve an important purpose in clinical care. The experience of empathy for the victim in response to observed injustice has been found to motivate observers of injustice to help the victim [30]. Taken together, this previous research suggests that clinicians experiences of vicarious injustice may depend on clinicians’ perceptions of the compensation system and their empathy for patients, and might motivate them to right the wrongs they perceive in the system through their clinical care.

### Coping Strategies and Burnout

Previous research and models of stress and burnout have described the impact of different coping strategies on wellbeing and performance at work. Lazarus [33] coined the terms “problem-focused” and “emotion-focused” to describe coping strategies that aim to address the problem or source of stress, and strategies that aim to reduce the emotional experience of stress, respectively. Research indicates that both strategies are associated with reductions in stress and improved workplace functioning, and that when used in combination, they can promote emotional balance and resilience [34, 35].

Participants in the current study described both “problem-focused” and “emotion-focused” strategies to navigate the complexities and challenges of working with compensable patients. Problem-focused strategies included seeking further education and training, seeking mentoring and professional supervision. Participants described using these strategies to directly address stress associated with self-doubt. Using these problem-focused strategies helped to improve their feeling of competence and sense of personal accomplishment [33]. Consistent with this, research indicates that continuing professional development is associated with lower burnout rates among physicians [19, 36] and nurses [37]. In addition, Lennon and colleagues found that physiotherapists who had undertaken further study in mental health areas reported being more confident to recognise and assess psychological distress in their clinical work [16]. Mentoring is considered to be a problem-focused solution as through mentoring, mentee’s learn to analyse problems more critically and develop their problem-solving skills. Mentors also often share their own experiences and problem-solving strategies, which mentees can adapt and apply to their own situations [38].

Emotion-focused strategies employed by participants in the current study included seeking reassurance and support from colleagues. These strategies helped them to become comfortable with ambiguity and protect them from the emotional toll of working with highly distressed and complex patients. Consistent with this, previous research has found that clinicians who participate in mentoring and peer support programmes are less likely to experience burnout and report higher job satisfaction [39].

### Promoting Clinician Wellbeing in Compensable Settings

Previous research has characterised the experiences of clinicians working with patients in compensable settings as burdensome, stressful, and emotionally draining [6, 21, 22]. By contrast, some of the participants in the present study described working in this clinical setting as a source of wellbeing. In particular, they described experiencing several of the five pillars of Seligman’s PERMA model [40, 41] in their work with compensable patients: Positive emotion, Engagement, Relationships, Meaning and Accomplishment. Specifically, participants described deriving satisfaction from facilitating patients’ recovery under difficult circumstances, engagement with their work as evidenced by efforts to further develop their skills and knowledge, belonging and support from relationships with colleagues, meaning from the process of supporting individuals in their recovery, and a sense of personal accomplishment or mastery from using their advanced training to address complex patient needs.

The mental wellbeing of clinicians is associated with better patient outcomes. For example, research has shown that clinicians who are mentally and physically healthy are more likely to provide high- quality care [42]. Burnout and fatigue among healthcare providers are strongly associated with medical errors, reduced patient engagement and lower continuity of care [43]. Further, clinicians leaving the profession due to burnout or other frustrations can lead to workforce shortages. Clinicians in the current study identified several strategies to support their wellbeing. We can protect clinician wellbeing by ensuring that workplaces encourage peer mentorship, supervision and enables access to professional development.

## Limitations

The clinicians interviewed in this research were self-selected, and their personal interest in volunteering to participate in the study may reflect a higher level of engagement with their profession or specifically with this area of practice. Moreover, many of the clinicians who were invited to participate in the study had a high level of experience and further education and training in communication and pain management. For these reasons, the perspectives and experiences shared by participants in the current study may not necessarily be shared by all clinicians working with patients who have a compensable injury. We suspect that clinicians with less experience or training, or less mentoring and clinical support, may be less likely to experience meaning and mastery in their work in this clinical setting. In addition, the majority of clinicians in the current research were physiotherapists or other physical medicine clinicians and the majority were located in Victoria. Hence it is probable that the views shared in the current research are less representative of the views of clinicians from other disciplines (who may have different education and training) or clinicians from states other than Victoria.

The experiences of the researcher completing the interviews may have influenced the focus of the interviews, meaning that certain topics or themes may have been explored more or less due to underlying biases.

Furthermore, by recruiting clinicians who are currently treating compensable patients, as opposed to clinicians who previously worked in this context and chose to not to continue this work, we represent only the views and experiences of clinicians who are currently satisfied or at least currently coping with this work. It is possible that clinicians who have left the profession or choose not to work with compensable patients have different, less positive experiences. Future research to explore the experiences of clinicians who have withdrawn from delivering care to this population could provide greater insights into how best to provide support to this cohort of clinicians. We also note that none of our participants mentioned any involvement in systems advocacy; practitioners can exert some influence on insurance bodies through their professional associations or peak bodies, which are often called upon by insurers for advice. We do not know whether the experience of clinicians who have the opportunity to advocate for changes in the system feel more hopeful or optimistic about their work, but it would be interesting for future research to explore the experience of being an advocate in the system.

### Implications and Future Directions

The findings of the current study, together with consistent findings reported in previous research, indicate that there are several steps that individual clinicians and healthcare organisations can take to improve the experience of working with patients who have a compensable injury. Clinicians themselves can direct their continuing professional education towards training which will improve their ability to manage patient distress and navigate psychosocial complexity in recovery from injury. Healthcare organisations or clinics can take steps to develop and encourage supervision, peer mentoring and peer support, which may help to mitigate emotional exhaustion and self-doubt. This is especially important for professions which do not routinely include supervision as a mandatory component of continuing professional development such as physiotherapy, osteopathy and nursing. Additionally, developing stratified models of care where high risk patients are treated by more experienced clinicians who have the appropriate training may help to ensure clinicians work at an appropriate level that sees them neither out of their depth, nor underutilising their skills. Taking these steps is likely to benefit not only clinicians themselves, but may also help to maintian the clinical workforce for adequate care delivery. Additionally, Seligman’s PERMA model provided a framework to contextualise clinician motivation in challenging settings such as the delivery of care in compensable settings. Future research could incorporate models such as PERMA to better frame clinician motivation [40, 41]

## Conclusion

Clinicians working with injured people who have a compensation claim report that delivering care in this space is complex when compared to delivering care to private patients (with no compensation claim) for a variety of reasons, including patient distress and administrative challenges. All clinicians found complexity challenging, however while some clinicians found these experiences stressful, for other clinicians these challenges offered them an opportunity for developing mastery and meaning in their work. Access to advanced training and workplace relationships seemed to play an important role in clinicians’ responses to these challenges.

## Supplementary Information

Below is the link to the electronic supplementary material.

Supplementary file1 (DOCX 14 KB)

## Data Availability

The datasets generated during and/or analysed during the current study are available from the corresponding author on reasonable request.
