# Supplementary material for: Clinician Experiences of and Responses to the Challenges of Working with Patients in the Australian Compensation Setting
Source: J Occup Rehabil. 2024 Sep 1;35(3):641–53. doi: 10.1007/s10926-024-10232-9 (PMC12361309; doi:10.1007/s10926-024-10232-9)
Supplement: Supplementary file 1 — Supplementary file1 (DOCX 14 KB) [file 10926_2024_10232_MOESM1_ESM.docx]

**Appendix A**

**Interview guide, Clinician participants**

Can you tell me about the nature of the work that you do with insurance clients?

Are there any elements of working with insurance clients that you find are rewarding?

Are there any elements of working with insurance clients that you find challenging?

Can you describe the nature of the contact that you have with the insurer in the process of treating insurance clients?

Are there any elements of interacting with the insurer, their staff and their processes that work well for you as a clinician?

Are there any elements of interacting with the insurer, their staff and their processes that are challenging? How might these be improved?
